# Cerium Chloride Application Promotes Wound Healing and Cell Proliferation in Human Foreskin Fibroblasts

**DOI:** 10.3390/ma10060573

**Published:** 2017-05-24

**Authors:** Liza L. Ramenzoni, Franz E. Weber, Thomas Attin, Patrick R. Schmidlin

**Affiliations:** 1 Clinic of Preventive Dentistry, Periodontology and Cariology, Center of Dental Medicine, University of Zurich, Plattenstrasse 11, 8032 Zurich, Switzerland; liza.ramenzoni@zzm.uzh.ch (L.L.R.); thomas.attin@zzm.uzh.ch (T.A.); 2 Oral Biotechnology and Bioengineering, Division of Cranio-Maxilo-Facial and Oral Surgery, Center of Dental Medicine, University of Zurich, Plattenstrasse 11, 8032 Zurich, Switzerland; franz.weber@zzm.uzh.ch

**Keywords:** cerium chloride, fibroblast, rare earth element, cell viability, cell migration

## Abstract

This study investigated the effect of cerium chloride (CeCl_3_) on cell migration and gene expression of human foreskin fibroblasts (HFF). HFF were exposed to three different CeCl_3_ solutions (1%, 5% and 10%, *w*/*v* %) for three different time durations (1, 5 and 10 min). 72 h after exposure to CeCl_3_, cell viability was assessed by MTT test. A scratch-wounded assay determined the cell migration and the width of the wound, measured at 24 h. Gene expression patterns for cyclins *B1*, *D1* and *E1* were analyzed by RT-PCR (*p* < 0.05, *t*-test). The viability proliferation increased at 1- and 5-min exposures for all CeCl_3_ concentrations, in contrast to no treatment (*p* < 0.05 at 24 h). No influence of CeCl_3_ was found after 10 min. The scratch assay showed increased cell migration up to 60% at 1 and 5 min after 24 h at 5% and 10%. Cyclin *B1*, *D1* and *E1* all showed upregulation, confirming an increase in cell proliferation. This study demonstrates that exposure time and concentration of CeCl_3_ may have a positive effect on fibroblast viability and migration. Application of CeCl_3_ may be beneficial as a cell-stimulating agent leading to therapeutic tissue fibrosis or more resistant tissue around teeth, when warranted, during different periodontal therapies.

## 1. Introduction

Cerium is a rare earth metal (lanthanoids series) and is also known to show similarities to calcium, which allows for its replacement without substituting its function [1]. In the medical field, cerium oxalate was used as an antiemetic, especially in vomiting during pregnancy and kinetoses without a clarified mechanism of action [1]. Currently, cerium nitrate is used for the topical treatment of extensive burns, as it has antiseptic effects, but it is also used in anticancer, anti-inflammatory, and antiviral agents [2,3,4]. In dentistry, cerium has also been used since 1975, when cerium nitrate was shown to potentially reduce the solubility of rat enamel [5] and a preventive effect of cerium was shown in human experimental root surfaces on carious-like lesions [6]. Wegenhaupt and co-workers recently showed that the application of cerium chloride could improve the acid resistance and anti-erosive potential of dentine [7,8].

One possible hindrance to the use of cerium is cell response to rare earth metals. Despite its low toxicity rate, some studies have shown this to be of concern [9,10,11]. For a chemical material to be considered as cyto- or biocompatible, both structure and function of the host tissue in direct contact with the agent need to be taken into consideration. In clinical studies, a negative dose-related effect of cerium on endocardial endothelial and cardiac fibroblast proliferation, as well as some pulmonary toxicity has been observed [9,10,11]. 

On the other hand, when testing the topical application of cerium in the oral cavity, Zhang et al. showed the protective effects of different lanthanoids, including cerium solutions, to dental hard tissues [6]. The same authors demonstrated that cerium influences the proliferation, differentiation, adipocytic transdifferentiation, and mineralization function of primary osteoblasts, depending on concentration and culture time [12]. Nair and co-workers showed a variation in the mitogenic response of cardiac and pulmonary fibroblasts to cerium and found that low levels stimulated the mitogenic response of fibroblasts [13]. 

Wound healing is a complex tissue remodeling process that entails several interactions among injured cells, extracellular matrix and factors involving inflammatory responses [13]. Cell proliferation and migration are known to play a ubiquitous role throughout the course of the tissue wound repair process [14]. Based on previous studies showing the use of cerium as a potential trigger of cell proliferation [15], it is reasonable to assume that this element could also play a role in wound healing-related tissue remodeling. Cerium chloride had been proven to possess cell proliferative properties in our previous in vitro study on osteoblast and human foreskin fibroblasts after short-term cell exposure to high concentrations of CeCl_3_ ranging from 10 mM to 100 mM [15]. Therefore, using the same in vitro study approach, we were interested in setting forth our line of investigation to include the possible effects of long term exposure to high concentrations of cerium on fibroblasts. We also sought to address the mechanisms underlying the cell wound-healing response to cerium chloride, as this biological aspect still remains unclear. 

The aim of this study was to test the cytocompatibility and dose-time-dependence of cerium chloride using a fibroblast model, which has already been used in comparable studies [13,15]. In addition, we focused on different genes expression patterns related to cell proliferation to evaluate the effect of cerium chloride on wound healing. Furthermore, the presumed positive effect was compared to a previously suggested proliferative effect of cerium solution in vitro [15]. We hypothesized that CeCl_3_ may act as a cell-stimulating agent in guided tissue fibrosis, which could clinically lead to more resistant tissues around teeth.

## 2. Results

### 2.1. Cell Viability

**Figure 1:**
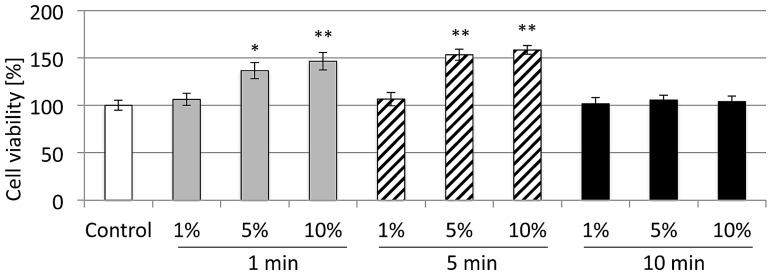
Human foreskin fibroblast (HFF) cell viability three days after CeCl_3_ exposure. A significant increase of cellular activity was seen for 1%, 5% and 10% solutions after both 1 and 5 min of exposure (* *p* < 0.05, ** *p* < 0.001, mean ± SD).

The results of the MTT test showed that human foreskin fibroblast (HFF) cell viability was significantly enhanced after 1 and 5 min of exposure to 1%, 5% and 10% CeCl_3,_ as compared to the cells cultured without CeCl_3_ (*p* < 0.05 at 24 h) exposure. No change was found after 10 min of exposure (Figure 1). HFF showed a significant increase in cellular activity three days after cerium exposure and up to 10% concentration levels.

### 2.2. Scratch-Wound Healing Assay

**Figure 2:**
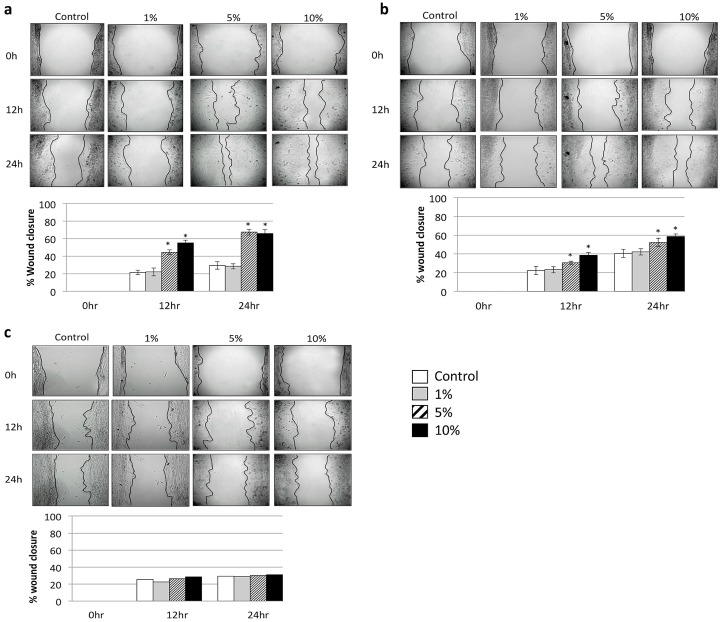
Induction of cell migration on in vitro scratch-wound healing assay after cerium exposure. (**a**) 1 min cerium treatment; (**b**) 5 min cerium treatment; (**c**) 10 min cerium treatment. (* *p* < 0.05, mean ± SD).

Cells exposed to cerium at concentrations of 5% and 10% showed an increase in cell migration up to 60% after CeCl_3_ exposure for 1 and 5 min on the scratch-wound healing assay at 24 h (Figure 2a,b). Consequently, wound closure was almost complete at 24 h in the presence of cerium. Controls, in contrast, showed no or incomplete healing patterns. No increase was found at 10 min of exposure (Figure 2c). 

### 2.3. Gene Expression Assay

**Figure 3:**
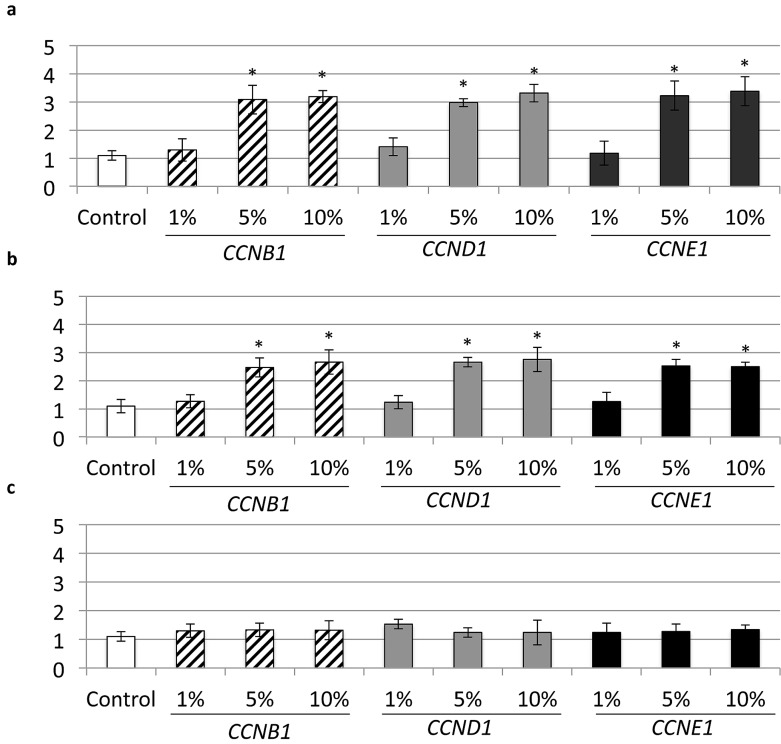
Cell proliferation genes *CCNB1*, *CCND1* and *CCNE1* analyzed by quantitative RT-PCR. (**a**) 1 min cerium treatment; (**b**) 5 min cerium treatment; (**c**) 10 min cerium treatment. (* *p* < 0.05, mean ± SD).

qPCR analysis showed an up-regulation of *CCNB1*, *CCND1* and *CCNE1* at the same concentrations (i.e., at 5% and 10%), which confirmed an increase in cell proliferation. This may facilitate wound healing and the cell migration process (Figure 3a,b). A time-dependent increase of *CCNB1*, *CCND1* and *CCNE1* expression was also evident on all cell cultures at 72 h after exposure to cerium. No increase in gene expression was found with 10 min of cerium exposure (Figure 3c).

## 3. Discussion

The aim of the present study was to investigate the influence of cerium chloride using cell migration/wound healing, proliferation and viability tests for fibroblasts. Different concentrations of lanthanum and cerium solutions application have been indicated to promote a shielding effect against erosion on dental hard tissues, such as the structure of crystal hydroxyapatite and its derivates [7,8,12]. Due to its mineralization properties, cerium formulations could potentially be advantageous for application on dental hard tissues. However, its use would lead to the direct exposure of surrounding soft tissue and oral mucosa. Since a few previous studies demonstrated a negative dose-related effect of cerium on endocardial endothelial proliferation of cardiac fibroblasts and even pulmonary toxicity [10,11], validation of the concentration of cerium related to toxic effects in soft tissues is required. To date, no single study has investigated the behavior of in vitro cells after being exposed to cerium chloride for different periods of time and what its implications would be for wound healing. 

Human foreskin fibroblasts were used in this study as they represent a relevant cell type in the field of dental research commonly applied in cytocompatibility tests [15,16]. In our previous study, cerium was confirmed to possess cell proliferative properties on osteoblast and human foreskin fibroblasts in vitro when exposed for short time periods [15]. Ten seconds of exposure was used as an attempt to mimic clinical rinsing conditions normally applied during periodontal treatment [16]. For the present study, longer exposure times were selected, i.e., of 1, 5 and 10 min. We found that cells were not negatively affected by cerium chloride application in terms of survival or activity. In contrast, after an exposure to 10% cerium, a significant increase in cellular activity and cell proliferation was detectable after 72 h. These results confirm previous results by Preeta and Nair, who showed a stimulation of cardiac fibroblast proliferation by exposure to low levels of cerium [17]. Different cell types may also react differently to cerium exposure, presenting diverse effects of cytotoxicity [18].

The effects of cerium on the proliferation, differentiation, and mineralization of mouse osteoblasts were investigated by Zhang and co-workers, where lower cerium concentrations were used [12]. In the present study, changes derived from short- to longer-term exposure and higher cerium concentrations were also assessed. In accordance with previous studies, our findings suggested that the effects similarly depended on the concentration, exposure and culture time. In order to ascertain the stimulation of fibroblast proliferation, we looked into the gene expression of cyclins *CCNB1*, *CCND1* and *CCNE1* as markers for cell proliferation and cell cycle [19]. The important association of cyclin *D1* and tissue regeneration has been noted by Ayala et al. [20]. In addition, this was correlated with a significant increase in cyclin *D1* expression, demonstrating that cell proliferation is affected at the level of G1/S cell cycle transition during proliferation [21]. Expression of cyclin *E1* and *B1*, which also mediate cell-cycle progression, were enhanced in dermal fibroblasts as wound healing progressed [22,23]. In our study, we found similar increases in gene expression of *CCNB1*, *CCND1* and *CCNE1* at up to 10 min of cerium exposure (Figure 3). Certain concentrations of rare earth compounds may inhibit the growth of cells or induce them to apoptosis by reducing cell cycle, however, it was also shown that there were no significant inhibitory effects on other cell lines [24]. At this point, we cannot conclude the reason why there was no higher expression of cyclins at 10 min of cerium exposure. Nevertheless, it has been already reported that only low concentrations of cerium improved mitotic response [13]. The mechanism of cell arrest needs to be further investigated. 

Another important result of the present study was the elucidation of cerium wound healing characteristics. Impaired wound healing and its medical complications in the field of periodontology results in a large burden for treatment success. The proliferation and migration of fibroblasts and keratinocytes primarily control wound healing and consequently will restore the normal skin composition [25]. In this sense, the use of rare earth elements as cell-stimulating agents could be accepted, as other authors have shown that cerium and cerium oxide nanoparticles potentially induce fibroblast and osteoblast proliferation and differentiation [12], skin wound healing [26] and even lead to a down-regulation of tumor growth and invasion [27].

With the objective of explaining the role of cerium as a mitogenic factor, many other authors sought to evaluate cell proliferation under different cerium concentrations. Kumar et al. confirmed that low doses of CeCl_3_ stimulated collagen and non-collagen protein synthesis in cardiac fibroblasts in vivo [28]. In fact, different low levels of cerium were shown to stimulate the mitogenic response of cardiac fibroblasts, but in a tissue-type dependent manner [13]. Interestingly, the same author indicated that the proliferative response was associated with intracellular generation of reactive oxygen species [13]. In addition, Preeta and Nair suggested that CeCl_3_-stimulated cell proliferation is possibly mediated by an early formation of superoxide-promoting growth responses [17]. These findings could corroborate the hypothesis of the decrease in cell proliferation and/or loss of viability observed after only 10 min of exposure at 1% in our results, as the proliferation is found in early formation of the superoxide anion-mediated response. Recently, a two-step free radical process in primary cultures of human foreskin exposed to cerium nanoparticles was reported [29,30]. In those studies, cerium exposure stimulated NADPH oxidase and a rapid increase in glutathione at non-cytotoxic/non-apoptotic doses, and also stimulated the induction of oxidative stress at doses higher than 0.1 g/L, which induced a 30–40% loss of mitochondrial activity. Overall, the difference in cell response found in our results due to short or longer exposure to cerium may be explained in part by early oxygen-derived free radical formation in parallel to cell proliferation.

We demonstrated a positive CeCl_3_ effect on the proliferation and wound healing response of fibroblasts, depending on the concentration and cell culture time. We could speculate that the use of cerium as a cell-stimulating agent may be possible for desired tissue fibrosis and, at the same time, the promotion of the calcification process in muco-gingival periodontal therapy or a resistant soft tissue on guided tissue regeneration. However, the implementation of the topical application of cerium that could accelerate wound healing merits further investigation i.e., in an animal model to provide a rational in order to develop this technology for use in humans.

## 4. Materials and Methods

### 4.1. Cerium Solutions

The stock solution of 10% CeCl_3_ (*w*/*v*) was prepared with 10 g CeCl_3_ (Sigma–Aldrich, Buchs, Switzerland) dissolved in 100 mL (end volume) of bidistilled sterile filtered water, yielding a final concentration of 270 mM. The CeCl_3_ solutions of 1% and 5% were prepared by dilution with sterile bidistilled water. The complexometric EDTA (Ethylenediaminetetraacetic acid) revealed the Ce^3+^ fraction to be 38.53%, yielding an effective Ce^3+^ of 101.8 mM for the 10% solution and 50.5 mM and 10.1 mM for the 5% and 1% solutions, respectively [15].

### 4.2. Cell Culture

Human foreskin fibroblasts (HFF) were a gift from Prof. Franz E. Weber (Center of Dental Medicine, University of Zurich) and maintained in Dulbecco’s Modified Eagle Medium (DMEM) (Invitrogen/Gibco, Zug, Switzerland) supplemented with 10% fetal bovine serum (FBS; 100 U/mL penicillin/Streptomicin and 1 g/L glucose). Medium was changed every three to four days and cells were passaged once a week. The cells used in this study were between the fifth and fifteenth passage. When confluent, the monolayers were detached with trypsin–EDTA for 10 min at 37 °C, centrifuged and washed with culture medium. 1 × 10^5^ cells were re-cultured in 6-well plates and grown until confluent monolayers were obtained again for 48 h. Then, 1 mL CeCl_3_ solutions of 1%, 5%, and 10% concentrations prepared in phosphate buffered saline (PBS) were added after the removal of the medium, respectively. After an exposure time of 1, 5, or 10 min, the CeCl_3_ solutions were aspirated and the cells washed with 1× PBS before the culture medium was newly added. At a selected time point of 72 h after exposure to the CeCl_3_ solutions, cell viability and gene expression were assessed. Cell migration (scratch-wound assay) was recorded until 24 h after wounding.

### 4.3. Cell Viability

HFF cell viability was determined by the thiazolyl blue tetrazolium (MTT; Sigma–Aldrich) dye reduction assay (5 mg/mL in phosphate buffered saline). HFF were grown in 6-well plates. At 72 h after exposure to the respective CeCl_3_ solutions, 500 mL of MTT was added to each well and incubated for 4 h at 37 °C in the dark. In the next step, MTT was removed by aspiration from the wells and isopropanol was added (200 mL; 1 N HCl) to solubilize the MTT-formazan crystals formed. The absorbance was measured at a wavelength of 570 nm with a plate spectrophotometer reader.

### 4.4. Scratch-Wound Assay (Cell Migration)

To determine the effect of CeCl_3_ solutions on wound healing, a scratch-wounded fibroblast monolayer model was used. HFF were plated into 6-well plate and cultured under serum starvation to a maximal 60% of confluence. The scratch was produced 16 h after the beginning of serum starvation of the cells, to halt proliferative response during wound closure. Next, each well was wounded by scratching with a 10 μL pipette tip. Following PBS washes to remove cell debris, the cultures were exposed to 1 mL CeCl_3_ solutions at different concentrations (1%, 5%, and 10% for 1 min, 5 min, and 10 min, respectively). Cell counting was also performed before and after the closure to assure a low change in cell number between the start and end of the experiment. Digital images were captured using a camera-equipped, inverted microscope (Carl Zeiss, Inc., Thorwood, NY, USA) and wound width measurements were subtracted from wound width at time zero to obtain the net wound closure. The distance between edges of the injured monolayer was measured by Image J software (NIH) in pixels and wound closure was expressed as the difference in width at 0 h, 12 h, and 24 h after wound simulation. The wound closure areas were measured with ImageJ (Software 1.48q, Rayne Rasband, National Institutes of Health, Bethesda, MD, USA) by subtracting the total amount of greyscale pixel counted in the cell-free area remaining after 24 h from the initial wound area (wound closure area). Since the scratch width varied to some extent from one wound to the other, a “relative wound closure” (RWC) area was calculated by normalizing the measured wound closure area (in pixels) to the total area of the image, which is covered in pixels (RWC (%) = wound closure area (pixel) × 100 (%)/× (pixel)).

### 4.5. Real-Time RT-PCR Analysis

Total RNA was isolated using TRIZOL reagent and an RNAeasy Mini kit (QIAGEN) 72 h after cerium exposure. Primer and probe sequences for genes encoding cyclin *B1* (*CCNB1*), *D1* (*CCND1*) and *E1* (*CCNE1*) were designed from Primer3 (version 0.4.0) (Table 1). Following TRIZOL extraction, real-time RT-PCR was performed using 15 mL final reaction volume of TaqMan’s One-step Master Mix kit (Applied Biosystems). Forty nanograms of the total RNA was used per sample well. Each sample contained pooled mRNA from TRIZOL extractions collected from the cell cultures exposed to 1 mL CeCl_3_ solutions of 1%, 5%, and 10% at respective time points (1 min, 5 min, and 10 min). All samples were tested in triplicates and three independent experiments were performed. The ∆Ct method was used to calculate gene expression levels relative to GAPDH and normalized to control cells (with no cerium).

### 4.6. Statistical Analysis 

The analysis of variance (ANOVA) was used for significant differences in results followed by the post hoc Fisher least significant difference (LSD) test. The paired two-tailed *t*-test was used to compare individual groups with each other. Statistical significance was set at *p* < 0.05. Statistical analysis was performed using the statistical software package SPSS 22.0 software for Windows and the values are shown with the median ± standard deviations from three different experiments, each one performed in triplicate.

## 5. Conclusions

Within the scope of this study, we conclude:

- Cell viability of HFF was significantly enhanced after 1 and 5 min applications of 5% and 10% CeCl_3_ compared to the cells cultured without CeCl_3_ (*p* < 0.05 at 72 h), whereas no cell activity was found for an exposure time longer than 10 min.
- Cells exposed to cerium at concentrations of 5% and 10% showed an increase in cell migration up to 60% at 1 and 5 min of CeCl3 exposure on the scratch-wound healing assay at 24 h. Consequently, wound closure was almost complete after 24 h in the presence of cerium, whereas this effect was far from being complete in the control samples.
- qPCR analysis showed the upregulation of *CCNB1*, *CCND1* and *CCNE1* in same concentrations of 5% and 10%, which confirmed the increase in the cell proliferation rate. This could facilitate wound healing and the cell migration process. A time-dependent increase of *CCNB1*, *CCND1* and *CCNE1* expression was evident on all cell cultures at 72 h after exposure to cerium.

## Figures and Tables

**Table 1:** Overview of the genes tested in this study.

Gene	Primer Pair Sequence
*CCNE1*	(forward) 5′-GAAATGGCCAAAATCGACAG-3′ (reverse) 5′-TCTTTGTCAGGTGTGGGGA-3′
*CCND1*	(forward) 5′-ACAAACAGATCATCCGCAAACAC -3′ (reverse) 5′-TGTTGGGGCTCCTCAGGTTC-3′
*CCNB1*	(forward) 5′-GGGTGTGCTTTGAATTCTGACA-3′ (reverse) 5′-AGGAGTGGCGCCTTGGTAT-3′
*GAPDH*	(forward) 5′-GCTCTCTGCTCCTCCCTGTT-3′ (reverse) 5′-CACACCGACCTTCACCATCT-3′

## Abbreviations

The following abbreviations are used in this manuscript:

CeCl_3_	cerium chloride
HFF	human foreskin fibroblasts
